# Personalized 3D-printed osteotomy guide combined with an ultrasonic bone scalpel for precise periapical cystectomy: a case report

**DOI:** 10.3389/fsurg.2026.1853640

**Published:** 2026-10-05

**Authors:** Jieying Li, Haoyu Sun, Xiaoyang Shan, Zhimin Zhang, Huibin Sun, Kai Zhou

**Affiliations:** 1 Department of Oral & Maxillofacial Surgery, The Affiliated Hospital of Qingdao University, Qingdao, Shandong, China; 2 School of Stomatology, Qingdao University, Qingdao, Shandong, China; 3 Department of Stomatology, The Affiliated Hospital of Qingdao University, Qingdao, Shandong, China

**Keywords:** 3D-printed osteotomy guide, apical surgery, case report, periapical lesions, ultrasonic bone scalpel

## Abstract

Periapical lesions persisting after root canal treatment, particularly those radiographically suggestive of true cysts, often necessitate surgical management. This report introduces a synergistic technique for precise periapical surgery in anatomically challenging areas, combining a 3D-printed osteotomy guide with an ultrasonic bone scalpel. A personalized, tooth-supported osteotomy guide was designed based on fused cone-beam computed tomography (CBCT) and intraoral scan data to ensure accurate localization of the lesion and minimal buccal bone window dimensions. An ultrasonic bone scalpel was then employed to execute the planned osteotomy, facilitating the reflection of a buccal bone lid that remained attached to the mucoperiosteal flap. Following complete cyst enucleation and apical resection, retrograde root canal filling was performed under microscopic vision. The pedicled bone lid was subsequently repositioned to promote healing and prevent soft tissue ingrowth. This combined protocol effectively addresses the challenges of limited visibility and access in the posterior maxilla, demonstrating a precise and minimally invasive approach for managing complex periapical lesions.

## 1 Introduction

Periapical lesions, frequently arising from persistent apical periodontitis, constitute a common clinical challenge in endodontics (1). While non-surgical root canal treatment (RCT) remains the cornerstone of management, a subset of cases necessitates surgical intervention (2). Established indications for periapical surgery include the failure of RCT evidenced by persistent symptoms or enlarging radiolucency, non-treatable root canal systems, large lesions suggestive of cysts, complex apical anatomy adjacent to vital structures such as the maxillary sinus or inferior alveolar nerve, and the need for histopathological confirmation (3, 4). Conventional periapical surgery, particularly in the posterior maxilla, is challenging due to limited surgical access, poor visibility, and risks associated with excessive bone removal or iatrogenic damage to adjacent anatomy, which may adversely affect outcomes and increase patient morbidity (5).

Technological advancements offer tools to address these difficulties. In the field of oral medicine, 3D printing technology is widely employed to create patient-specific anatomical models, design and fabricate highly accurate surgical guides, and finds broad application in procedures such as mandibular reconstruction surgeries, orthognathic surgeries, facial fracture, and dental implant treatments (6). In endodontic microsurgery, 3D-printed templates have been reported for guided osteotomy and root-end resection (7), “targeted” single-step osteotomy with trephine burs (8), and apex localization beneath a thick buccal bone plate (9); more recently, the “bone window” concept (10) has been combined with piezoelectric surgery, with or without a CAD/CAM-guided stent, to preserve and reposition the buccal cortical plate (11, 12). Separately, ultrasonic bone scalpels have gained traction for enabling selective, minimally invasive osteotomy with minimal thermal injury to adjacent tissues, as demonstrated in various complex oral surgical procedures (13). However, in most of these protocols the template serves mainly to localize the apex while the osteotomy is performed freehand, and harvested cortical lids are detached and stored *ex vivo* as free grafts; moreover, an integrated guided workflow for the posterior maxilla—where access is limited, the buccal plate is thin, and the maxillary sinus is at risk—has rarely been reported. We therefore combined a tooth-supported 3D-printed guide designed to delineate a minimal bone window matched to the segmented lesion with an ultrasonic bone scalpel, so that the buccal cortical lid remained pedicled on the mucoperiosteal flap and could be repositioned immediately after cyst enucleation, without *ex vivo* storage, bone substitutes, or barrier membranes.

The contribution of this report lies not in the use of a printed guide or an ultrasonic device *per se*, but in their integration into a single workflow in which the guide delineates a lesion-matched minimal bone window and the ultrasonic osteotomy preserves a pedicled buccal bone lid for immediate repositioning. We describe the application of this integrated digital-microsurgical approach in managing a persistent periapical lesion associated with a maxillary first molar, in which the synergy between digital planning and ultrasonic execution enabled a pre-planned fenestration, limited bone removal, intentional preservation of the bone lid, and favorable healing. This protocol may provide a practical reference for minimally invasive periapical surgery in anatomically demanding regions.

## 2 Materials and methods

### 2.1 Case selection and preoperative assessment

A 35-year-old male patient presented to our clinic with persistent discomfort in the maxillary right posterior region. The maxillary right first molar had undergone conventional root canal treatment of all three roots before referral and was restored with a direct composite resin restoration; the tooth elicited discomfort on percussion, while palpation was unremarkable, and there was no sinus tract, no abnormal mobility, and periodontal probing depths of approximately 2 mm; the adjacent teeth responded normally to pulp vitality testing. Periapical radiography and cone-beam computed tomography (CBCT) revealed a well-defined, unilocular radiolucent lesion measuring approximately 5.5 × 6.5 mm, confined to the apex of the mesiobuccal root of this three-rooted tooth, with no communication with the apices of the other roots and a bony separation of approximately 2 mm from the maxillary sinus floor, consistent with symptomatic apical periodontitis and a suspected periapical cyst (Figure 1). Follow-up CBCT obtained after a 3-month observational period revealed persistence and enlargement of the lesion (Figure 2), indicating failure of non-surgical management and necessitating surgical intervention. All CBCT examinations were justified in accordance with the AAE/AAOMR and European Society of Endodontology position statements on the use of CBCT in endodontics (14, 15) and the SEDENTEXCT guidelines (16): the preoperative scan was required for three-dimensional evaluation of the lesion and provided the DICOM data necessary for guide design, and the 3-month scan documented lesion progression after failed root canal treatment, in line with the ALARA (as low as reasonably achievable) principle; notably, the preoperative dataset served both diagnostic evaluation and guide design, so that no additional scan was required for planning purposes.

**Figure 1:**
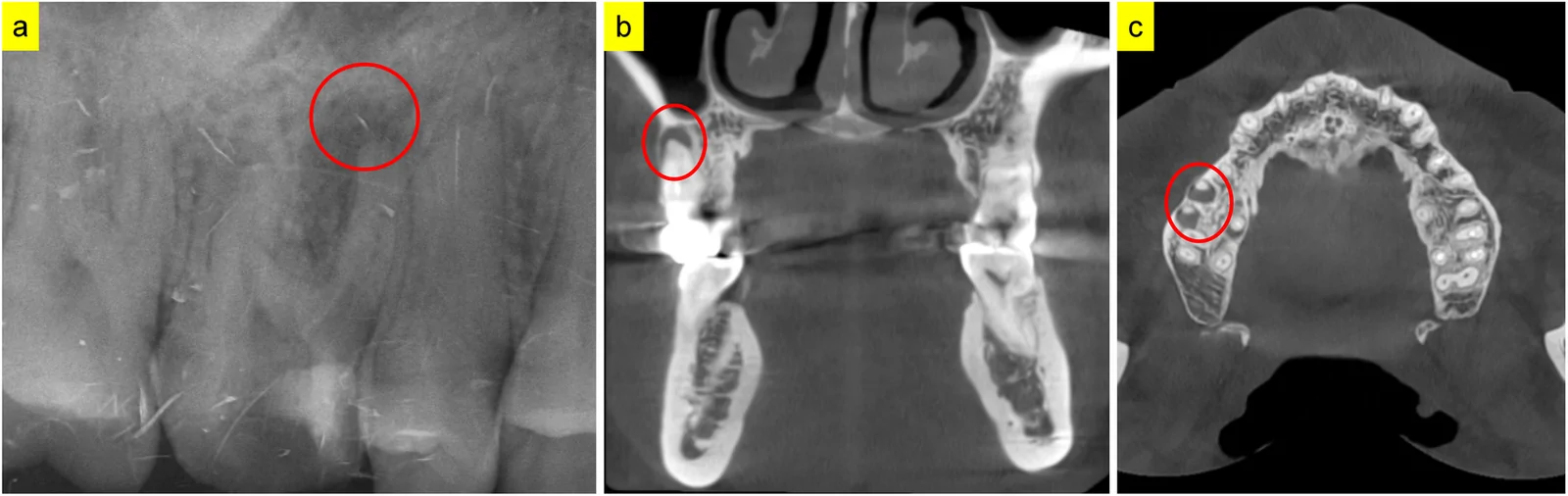
Before root canal treatment imaging revealing a periapical radiolucent lesion associated with the maxillary first molar. **(a)** Intraoral periapical radiograph demonstrating a well-defined, low-density radiolucent area at the apical region of the maxillary right first molar. **(b)** Coronal section of cone-beam computed tomography (CBCT) showing a round, unilocular radiolucency with corticated borders involving the apical region of the same tooth. **(c)** Axial CBCT view confirming the three-dimensional extent of the lesion, with no evidence of cortical expansion or root resorption.

**Figure 2:**
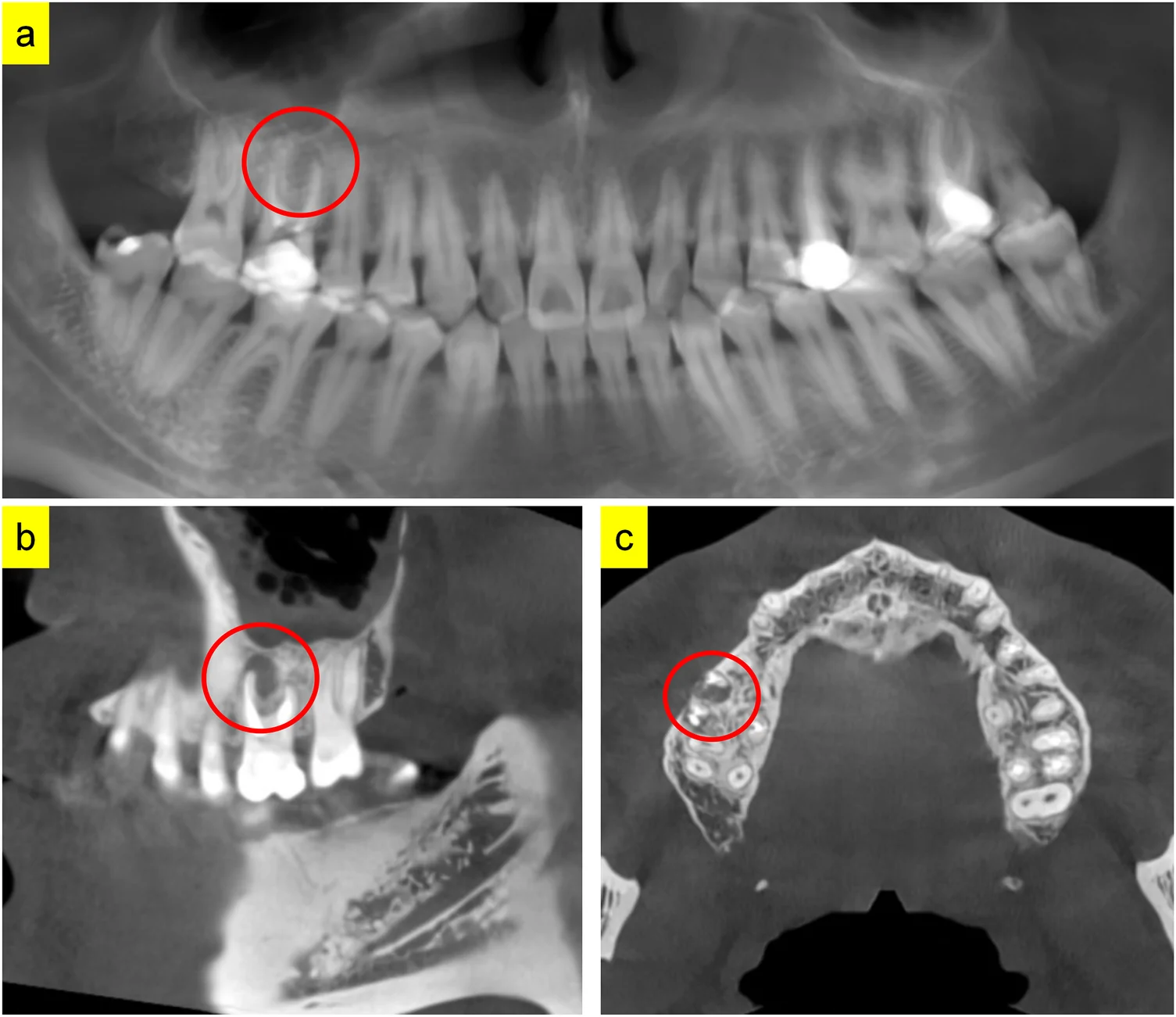
Follow-up imaging at 3 months after root canal treatment showing persistent and progressive radiolucency in the apical region of the maxillary first molar. **(a)** Panoramic radiograph revealing an enlarged, well-defined radiolucent lesion associated with the apex of the maxillary right first molar. **(b)** Sagittal CBCT section demonstrating increased lesion size with preservation of cortical margins and no signs of bone expansion or perforation. **(c)** Axial CBCT view confirming the three-dimensional enlargement of the periapical lesion, with continued absence of internal septations or calcifications.

### 2.2 Digital planning and surgical guide fabrication

Surgical planning followed a fully digital workflow. The DICOM data from the CBCT scan (Planmeca ProMax 3D, Planmeca Oy, Helsinki, Finland; field of view Ø15.4 × 8.1 cm; voxel size 0.2 mm; 90 kV, 7.1 mA, exposure time 12.4 s) and the STL data acquired with an intraoral scanner (3Shape TRIOS; TRIOS software version 20.1.2, 3Shape, Copenhagen, Denmark) were imported into dedicated planning software (SRP1902A, SprintRay, China) (Figures 3a–d) and co-registered by a surface-matching algorithm based on the dental contours; the accuracy of co-registration was verified by visual inspection of anatomical landmark superimposition. The boundaries of the periapical lesion were segmented semi-automatically (threshold-based segmentation with manual refinement) to visualize its spatial relationship with the root apex and the maxillary sinus. A tooth-supported osteotomy guide (thickness 2.0 mm; offset 0.05 mm) was then designed, with the osteotomy window dimensioned to the segmented lesion (5 × 5 mm; Figures 3a,b). The guide was fabricated on a digital light processing printer (SprintRay Pro 95, SprintRay China, Zhejiang Xunshi Technology Co., Ltd., China) at a layer thickness of 100 µm, with the intaglio surface oriented away from the build platform, using an autoclavable dental surgical guide resin (SP-RB0808, SprintRay China). After printing, the guide was washed in isopropyl alcohol, ultraviolet post-cured, and sterilized by autoclaving according to the manufacturer's protocol. Dimensional quality control comprised verification of passive fit on the working model and intraorally, together with measurement of the window dimensions against the design values.

**Figure 3:**
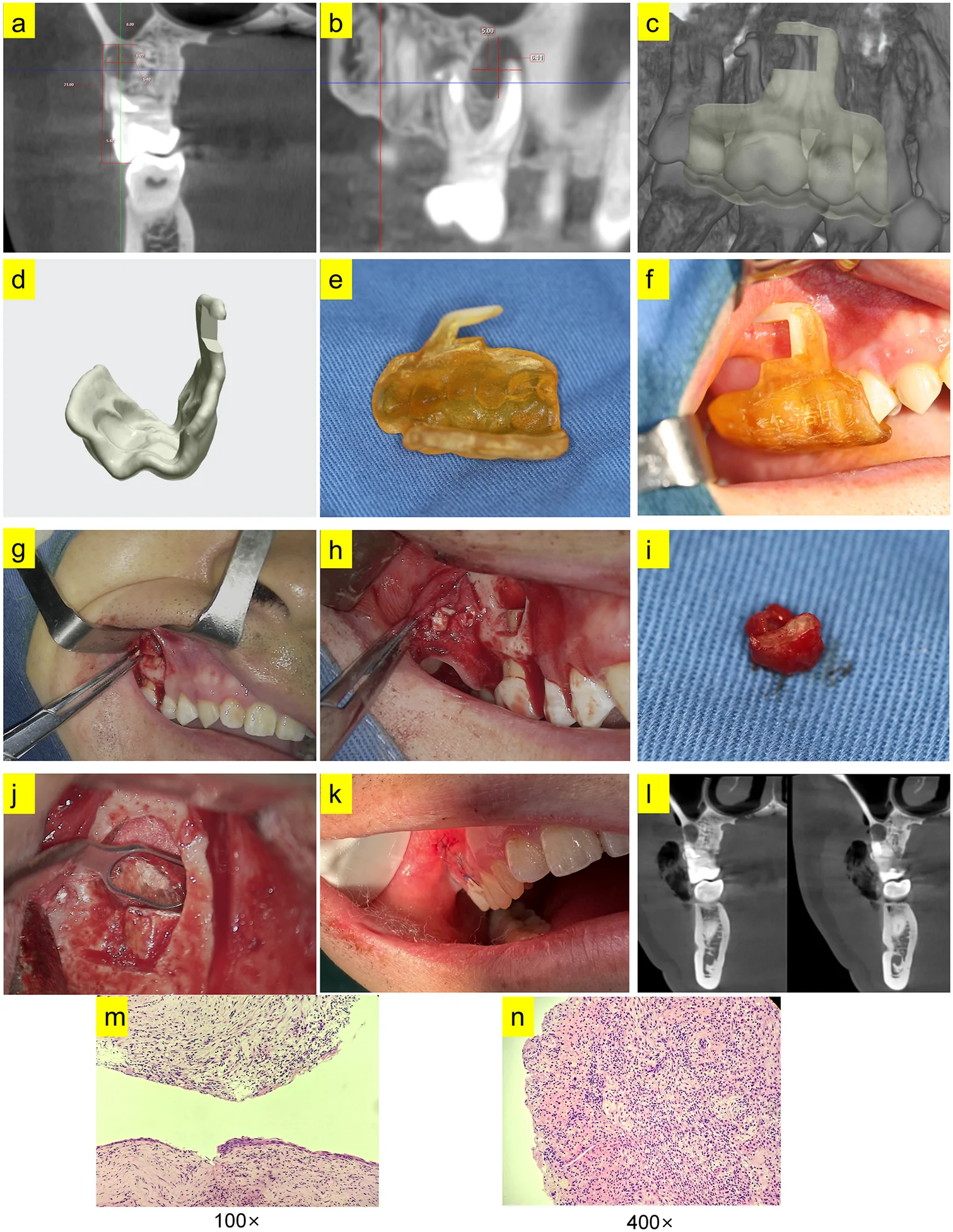
Step-by-step surgical workflow for precise periapical cyst enucleation using a 3D-printed osteotomy guide and an ultrasonic bone scalpel. **(a,b)** Preoperative cone-beam computed tomography (CBCT) slices used for virtual measurement and planning of the osteotomy window. **(c)** Three-dimensional reconstruction of the maxillary dental arch from CBCT data, serving as the basis for digital guide design. **(d)** Computer-aided design (CAD) model of the customized osteotomy guide. **(e)** 3D-printed surgical guide fabricated from an autoclavable dental surgical guide resin (SP-RB0808, SprintRay) using a digital light processing printer (SprintRay Pro 95). **(f)** Intraoperative seating of the guide for localization and stabilization during osteotomy. **(g)** Reflection of the pedicled buccal bone lid together with the mucoperiosteal flap, exposing the lesion. **(h)** Root-end resection of the mesiobuccal root apex within the bony window. **(i)** Excised cystic specimen showing an intact capsule. **(j)** Microscopic retrograde root canal filling with a premixed calcium-silicate–based bioceramic root repair material (iRoot BP Plus) after root-end preparation. **(k)** Repositioning of the bone lid and primary closure of the flap. **(l)** Immediate postoperative CBCT demonstrating complete removal of the lesion, correct positioning of the repositioned bone lid, and preservation of surrounding bone architecture. **(m)** Low-power magnification (×100) revealing a well-defined cystic structure lined by stratified squamous epithelium, with a fibrous connective tissue wall showing moderate chronic inflammatory infiltrate. **(n)** High-power magnification (×400) demonstrating dense infiltration of lymphocytes and plasma cells within the cyst wall, consistent with a diagnosis of radicular cyst. No evidence of atypia, dysplasia, or malignant transformation was observed (m, n: hematoxylin and eosin staining).

### 2.3 Surgical procedure

Under local anesthesia, a sulcular incision was made from the maxillary right second premolar to the second molar, with a vertical releasing incision mesial to the premolar. The surgical guide was seated on the teeth, and its stability was verified by the absence of any perceptible movement under manual pressure (Figure 3f). The tip of the ultrasonic bone scalpel (PIEZOTOME SOLO LED, SATELEC/ACTEON, France) was inserted through the guide window and advanced along its inner border at power setting 7 with light, intermittent strokes under continuous sterile saline irrigation, so that the guide mechanically constrained the position, outline, and angulation of the osteotomy; the cutting depth was controlled according to the preoperatively measured distance from the buccal cortical surface to the lesion (Figures 3a,b). The osteotomy was performed through the full thickness of the buccal cortical plate (approximately 1.0 mm) on three sides of the window, without elevating the mucoperiosteum from the cortical surface; the buccal cortical bone lid (approximately 4.5 × 4.5 × 1.0 mm; hereafter, “bone lid”) was then gently mobilized by controlled fracture along the remaining side and reflected together with the overlying mucoperiosteal flap, to which it remained pedicled (Figure 3g). The cyst was completely enucleated (Figure 3i). Histopathological examination confirmed a cystic lesion (Figures 3m,n). Subsequently, approximately 3 mm of the apex of the mesiobuccal root was resected with a bevel of approximately 10° to permit direct microscopic visualization (Figure 3h). After methylene blue staining, the resected root surface was examined under an operating microscope at × 16 – × 30 magnification to identify the canal anatomy, calcified canal orifices, isthmus, and any microcracks. Root-end preparation was then performed at × 8 – × 16 magnification using diamond-coated 90° ultrasonic retro-preparation tips (ASLD/ASRD, ACTEON Group, France) at power setting 7 under continuous saline irrigation, creating a cavity approximately 3 mm in depth along the long axis of the root (17). Hemostasis was achieved with epinephrine-impregnated cotton pellets packed into the bony crypt. After thorough drying of the cavity, a premixed calcium-silicate–based bioceramic root repair material (iRoot BP Plus, Innovative BioCeramix, Vancouver, Canada) was delivered with a dedicated carrier and compacted under microscopic vision to ensure a hermetic apical seal (Figure 3j). The bony cavity was packed with absorbable gelatin sponge and recombinant bovine basic fibroblast growth factor (rb-bFGF) gel, and copious irrigation with normal saline was carried out to eliminate any potential microscopic resin debris derived from the surgical guide. Finally, the pedicled bone lid was repositioned into the osteotomy window, where it was retained by close geometric adaptation to the window margins and by tension-free interrupted 4-0 non-resorbable sutures of the attached mucoperiosteal flap, without additional fixation devices or barrier membranes (Figure 3k). No clinically perceptible mobility of the bone lid was observed after suturing. A postoperative CBCT scan was obtained immediately to confirm the correct position of the repositioned bone lid and to exclude retained fragments or inadvertent communication with the maxillary sinus (Figure 3l). The achieved osteotomy window measured approximately 4.5 × 4.5 mm on the postoperative CBCT, closely matching the planned dimensions; the minor reduction is consistent with the kerf width of the ultrasonic tip.

### 2.4 Postoperative follow-up

The sutures were removed after one week, and postoperative healing was uneventful. Clinical and radiographic evaluations were conducted at the 1-year follow-up. The follow-up CBCT was considered justified, in accordance with the AAE/AAOMR and ESE position statements (14, 15), to evaluate three-dimensional healing of the former cystic cavity and integration of the repositioned bone lid, which cannot be reliably assessed on two-dimensional radiographs owing to anatomical superimposition, and was performed in line with the ALARA principle. For standardized comparison, the follow-up images were reoriented along the long axis of the treated tooth. Radiographic healing was assessed according to the criteria of Molven et al. (18) and the CBCT-based healing criteria described by von Arx et al. (19). The preoperative lesion (approximately 5.5 × 6.5 mm) showed complete radiographic resolution at 1 year, with radiographic fill of the former cystic cavity by bone-density tissue and no evidence of recurrence, corresponding to complete healing (Figure 4). It should be noted that CBCT demonstrates radiographic healing but cannot establish the histological nature of the regenerated tissue.

**Figure 4:**
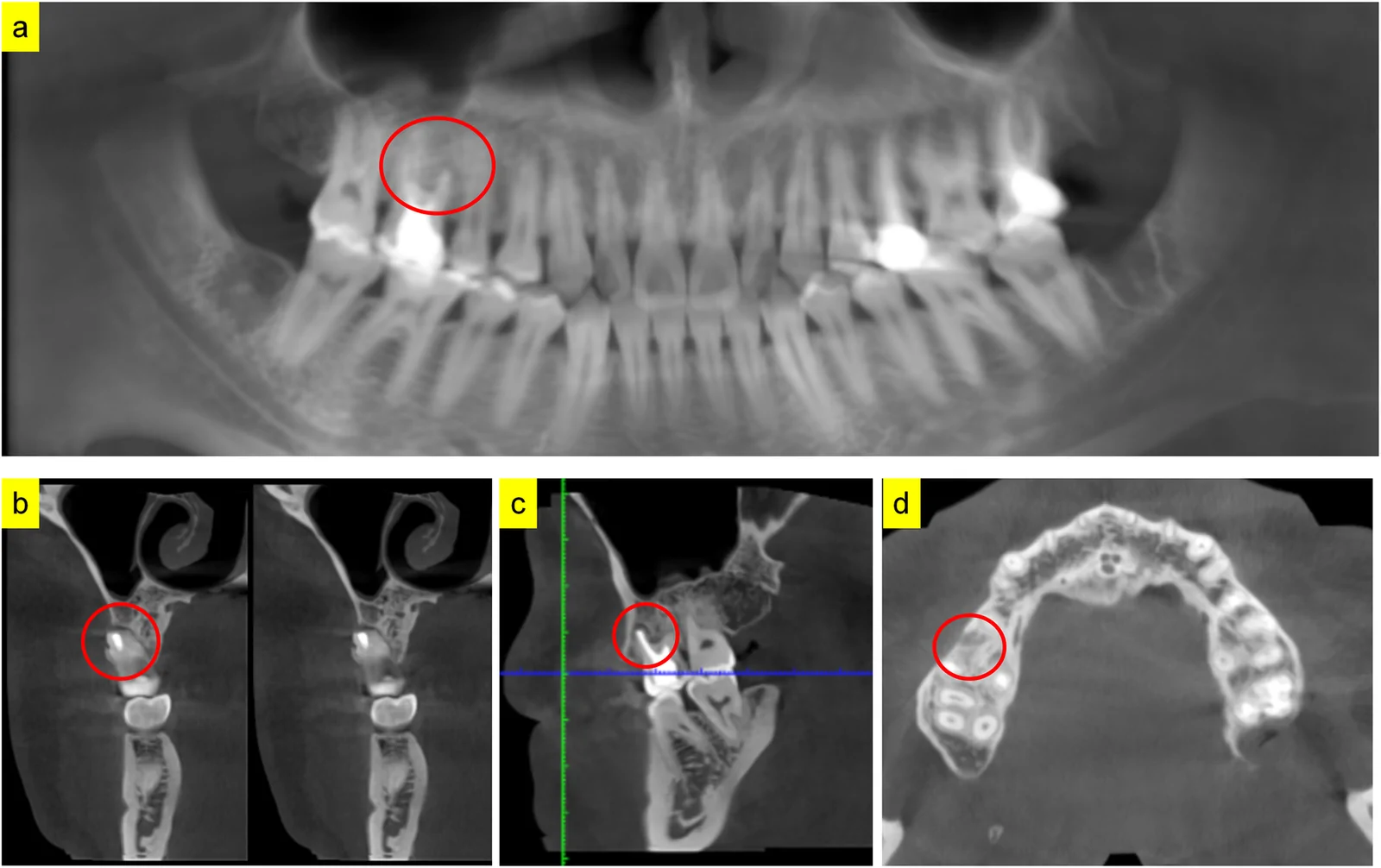
One-year postoperative follow-up imaging demonstrating radiographic healing of the periapical lesion. **(a)** Panoramic radiograph showing resolution of the previous radiolucency in the apical region of the maxillary right first molar. **(b)** Coronal CBCT sections showing radiographic fill of the former cystic cavity with bone-density tissue and no residual radiolucency. **(c)** Sagittal CBCT view showing no evidence of lesion recurrence and preservation of the alveolar bone contour. **(d)** Axial CBCT image showing radiographic fill of the surgical site. The nature of the regenerated tissue cannot be determined radiographically.

## 3 Discussion

Periapical cysts, a form of odontogenic jaw cyst arising from persistent chronic inflammation at the root apex, possess the potential to erode surrounding bone and compromise dental structures, leading to functional and aesthetic sequelae (20). This report presents a case in which conventional RCT failed to resolve periapical inflammation. A new treatment approach was implemented, combining a 3D-printed osteotomy guide with an ultrasonic bone cutting device for periapical surgery. The novelty of this protocol lies not merely in the sequential use of two advanced technologies, but in their deliberate synergy within a fully digital workflow to execute a pre-defined, minimally invasive surgical plan. This integrated technique enabled complete removal of the periapical lesion in this anatomically challenging case.

Although RCT remains the first-line treatment for apical periodontitis, the lack of clinical improvement after a three-month observation period in this case suggests potential contributing factors such as incomplete treatment, residual infection, complex root canal anatomy, or other impediments to cyst regression (21, 22). Under such circumstances, timely surgical intervention becomes crucial in managing periapical lesions, especially when conventional RCT is ineffective (3). Prioritizing the treatment of the root apex and the surrounding apical lesion can help eliminate the source of infection and promote cyst healing. The combined use of a patient specific 3D-printed osteotomy guide and an ultrasonic bone scalpel was intended to enhance intraoperative precision and safety while preserving native bone. In posterior regions, particularly the maxilla, conventional periapical surgery is often hindered by poor visibility, difficult access, and limited maneuverability. Even with guided surgery focused primarily on root end localization, osteotomies performed with dental fissure bur can generate heat and risk damaging the thin buccal bone plate (23). To overcome these limitations, our approach utilized a digitally planned, anatomically adapted 3D-printed osteotomy guide to establish a precise surgical pathway. This was expected to improve procedural predictability and reduce operative uncertainty—potential advantages that may be especially critical in the deep, spatially confined posterior maxilla. Such guides may reduce unnecessary bone removal and help preserve healthy periodontal tissues; however, in the absence of deviation measurements in this case, these effects remain presumptive. The ultrasonic bone knife, a minimally invasive tool for cutting bone, enables accurate bone tissue removal while it may reduce heat-related and mechanical damage to adjacent soft tissues (13, 24). In this case, it allowed for more precise bone incisions at the fenestration site, potentially contributing to reduced intraoperative bleeding and a clearer surgical field, a critical aspect in the deep and confined space of the maxillary posterior region. Additionally, the selective cutting characteristics of ultrasonic devices may help protect adjacent soft-tissue structures, which could lower the risk of postoperative complications (13). This precise osteotomy is the critical prerequisite for the subsequent intentional reflection and repositioning of the pedicled buccal bone lid. This technique may create a natural biological barrier against soft-tissue ingrowth, and in this case no barrier membrane was used. By preserving the cortical bone architecture and its periosteal blood supply, the repositioned bone lid may serve as an osteoconductive scaffold that supports bone healing from the periphery; however, the radiographic fill observed on the 1-year follow-up CBCT cannot establish the histological nature of the regenerated tissue. Following apical resection, retrograde microsurgical RCT was performed under surgical microscopy to ensure thorough cleaning and hermetic sealing of the apical region. This approach aims to prevent persistent infection and create favorable conditions for postoperative healing (25).

The present workflow should also be considered in the context of other computer-aided guidance technologies for endodontic microsurgery, namely dynamic navigation and robotic systems (26). Dynamic navigation provides real-time visual feedback and permits intraoperative modification of the surgical plan without a physical template; cadaver and *in vitro* studies have reported lower deviations in osteotomy and root-end resection compared with freehand surgery (27), and its feasibility has been demonstrated in posterior teeth (28). However, it entails a learning curve, continuous hand–eye coordination, line-of-sight tracking, and considerably higher equipment costs, and it provides no physical constraint on the instrument. Robotic assistance represents the most recent development: haptic-guided and autonomous robotic platforms have been reported for osteotomy and root-end resection (29, 30), and a comparative *in vitro* study found robot-assisted endodontic microsurgery to achieve the highest accuracy among static, dynamic, and robotic approaches, albeit with increased cost and system complexity (31). By contrast, static tooth-supported guides—such as the one used in this case—are inexpensive, rapidly fabricated, and require no intraoperative tracking hardware; however, the plan cannot be modified once the guide is printed, and accuracy depends on the entire data-acquisition, registration, and manufacturing chain. In the present case, stable occlusal support and a well-defined unilocular lesion favored a static guide, whereas scenarios requiring intraoperative plan changes or lacking stable dental support may benefit from dynamic or robotic guidance. Integration of ultrasonic osteotomy with dynamic navigation or robotic platforms warrants further investigation.

In conclusion, this case report demonstrates the feasibility of combining digital planning with a lesion-adapted, 3D-printed osteotomy guide and ultrasonic osteotomy for periapical surgery in the posterior maxilla. In this patient, the protocol was associated with a small, pre-planned fenestration, preservation and repositioning of a pedicled buccal bone lid, and complete radiographic resolution of the lesion at the 1-year follow-up. By minimizing iatrogenic bone removal and optimizing the conditions for bone regeneration, this technology approach may improve patient recovery and offers valuable clinical insights and practical experience for similar anatomically demanding cases. Whether it confers measurable advantages over conventional surgery, dynamic navigation, or robotic assistance cannot be determined from a single case and warrants controlled comparative studies.

## Data Availability

The original contributions presented in the study are included in the article/Supplementary Material, further inquiries can be directed to the corresponding authors.

## References

[1] Nascimento D Guedes IC Brand LM Bernardi L Vasconcelos JM Figueiredo JAP. Bacterial viability in persistent apical periodontitis: a systematic review. J Endod. (2025) 51(11):1549–56. 10.1016/j.joen.2025.07.015 40714054

[2] Artaza L Campello AF Soimu G Alves FRF Rôças IN Siqueira JF Jr. Outcome of nonsurgical root canal treatment of teeth with large apical periodontitis lesions: a retrospective study. J Endod. (2024) 50(10):1403–11. 10.1016/j.joen.2024.08.003 39151889

[3] Wang H Xu X Bian Z Liang J Chen Z Hou B. Expert consensus on apical microsurgery. Int J Oral Sci. (2025) 17(1):2. 10.1038/s41368-024-00334-8 39743567 PMC11693765

[4] Lieblich SE. Current concepts of periapical surgery: 2020 update. Oral Maxillofac Surg Clin North Am. (2020) 32(4):571–82. 10.1016/j.coms.2020.07.007 32912776

[5] Serrano-Giménez M Sánchez-Torres A Gay-Escoda C. Prognostic factors on periapical surgery: a systematic review. Med Oral Patol Oral Cir Bucal. (2015) 20(6):e715–22. 10.4317/medoral.20613 PMC4670252 26449431

[6] Figueiredo-Pina CG Serro AP. 3D printing for dental applications. Materials (Basel). (2023) 16(14):4972. 10.3390/ma16144972 37512251 PMC10381496

[7] Strbac GD Schnappauf A Giannis K Moritz A Ulm C. Guided modern endodontic surgery: a novel approach for guided osteotomy and root resection. J Endod. (2017) 43(3):496–501. 10.1016/j.joen.2016.11.001 28139285

[8] Giacomino CM Ray JJ Wealleans JA. Targeted endodontic microsurgery: a novel approach to anatomically challenging scenarios using 3-dimensional-printed guides and trephine burs-a report of 3 cases. J Endod. (2018) 44(4):671–7. 10.1016/j.joen.2017.12.019 29426644

[9] Ahn SY Kim NH Kim S Karabucak B Kim E. Computer-aided design/computer-aided manufacturing-guided endodontic surgery: guided osteotomy and apex localization in a mandibular molar with a thick buccal bone plate. J Endod. (2018) 44(4):665–70. 10.1016/j.joen.2017.12.009 29358006

[10] Khoury F Hensher R. The bony lid approach for the apical root resection of lower molars. Int J Oral Maxillofac Surg. (1987) 16(2):166–70. 10.1016/s0901-5027(87)80125-X 3110314

[11] Lee SM Yu YH Wang Y Kim E Kim S. The application of “bone window” technique in endodontic microsurgery. J Endod. (2020) 46(6):872–80. 10.1016/j.joen.2020.02.009 32312482

[12] Kim U Kim S Kim E. The application of “bone window technique” using piezoelectric saws and a CAD/CAM-guided surgical stent in endodontic microsurgery on a mandibular molar case. Restor Dent Endod. (2020) 45(3):e27. 10.5395/rde.2020.45.e27 32839708 PMC7431938

[13] Stübinger S Landes C Seitz O Zeilhofer HF Sader R. Ultrasonic bone cutting in oral surgery: a review of 60 cases. Ultraschall Med. (2008) 29(1):66–71. 10.1055/s-2007-963507 18270888

[14] Special Committee to Revise the Joint AAE/AAOMR Position Statement on use of CBCT in Endodontics. AAE and AAOMR joint position statement: use of cone beam computed tomography in endodontics 2015 update. Oral Surg Oral Med Oral Pathol Oral Radioll*.* (2015) 120(4):508–12. 10.1016/j.oooo.2015.07.033 26346911

[15] Patel S Brown J Semper M Abella F Mannocci F. European society of endodontology position statement: use of cone beam computed tomography in endodontics: european society of endodontology (ese) developed by. Int Endod J. (2019) 52(12):1675–8. 10.1111/iej.13187 31301231

[16] European Commission. Radiation Protection No. 172: Cone Beam CT for Dental and Maxillofacial Radiology. Evidence-Based Guidelines. Luxembourg: European Commission (2012).

[17] Kim S Kratchman S. Modern endodontic surgery concepts and practice: a review. J Endod. (2006) 32(7):601–23. 10.1016/j.joen.2005.12.010 16793466

[18] Molven O Halse A Grung B. Observer strategy and the radiographic classification of healing after endodontic surgery. Int J Oral Maxillofac Surg. (1987) 16(4):432–9. 10.1016/s0901-5027(87)80080-2 3117915

[19] von Arx T Janner SF Hänni S Bornstein MM. Agreement between 2D and 3D radiographic outcome assessment one year after periapical surgery. Int Endod J. (2016) 49(10):915–25. 10.1111/iej.12548 26356580

[20] Rajendra Santosh AB. Odontogenic cysts. Dent Clin North Am. (2020) 64(1):105–19. 10.1016/j.cden.2019.08.002 31735221

[21] Nair PN. Pathogenesis of apical periodontitis and the causes of endodontic failures. Crit Rev Oral Biol Med. (2004) 15(6):348–81. 10.1177/154411130401500604 15574679

[22] Tabassum S Khan FR. Failure of endodontic treatment: the usual suspects. Eur J Dent. (2016) 10(1):144–7. 10.4103/1305-7456.175682 27011754 PMC4784145

[23] Rashad A Sadr-Eshkevari P Heiland M Smeets R Hanken H Gröbe A. Intraosseous heat generation during sonic, ultrasonic and conventional osteotomy. J Craniomaxillofac Surg. (2015) 43(7):1072–7. 10.1016/j.jcms.2015.05.018 26116310

[24] Wang Q Zhang T. Application of an ultrasonic bone knife combined with a dental electric motor in the extraction of mandibular middle and low impacted teeth. BMC Oral Health. (2024) 24(1):18. 10.1186/s12903-023-03788-0 38178077 PMC10768189

[25] Alghamdi F Alhaddad AJ Abuzinadah S. Healing of periapical lesions after surgical endodontic retreatment: a systematic review. Cureus. (2020) 12(2):e6916. 10.7759/cureus.6916 32190471 PMC7061768

[26] Ribeiro D Reis E Marques JA Falacho RI Palma PJ. Guided endodontics: static vs. dynamic computer-aided techniques-a literature review. J Pers Med. (2022) 12(9):1516. 10.3390/jpm12091516 36143301 PMC9501573

[27] Aldahmash SA Price JB Mostoufi B Griffin IL Dianat O Tordik PA. Real-time 3-dimensional dynamic navigation system in endodontic microsurgery: a cadaver study. J Endod. (2022) 48(7):922–9. 10.1016/j.joen.2022.04.012 35489425

[28] Fu W Chen C Bian Z Meng L. Endodontic microsurgery of posterior teeth with the assistance of dynamic navigation technology: a report of three cases. J Endod. (2022) 48(7):943–50. 10.1016/j.joen.2022.03.010 35398147

[29] Isufi A Hsu TY Chogle S. Robot-assisted and haptic-guided endodontic surgery: a case report. J Endod. (2024) 50(4):533–9.e1. 10.1016/j.joen.2024.01.012 38280513

[30] Liu C Liu X Wang X Liu Y Bai Y Bai S. Endodontic microsurgery with an autonomous robotic system: a clinical report. J Endod. (2024) 50(6):859–64. 10.1016/j.joen.2024.02.005 38369101

[31] Chen C Qin L Zhang R Meng L. Comparison of accuracy and operation time in robotic, dynamic, and static-assisted endodontic microsurgery: an *in vitro* study. J Endod. (2024) 50(10):1448–54. 10.1016/j.joen.2024.05.018 38838934

